# Sugarcane Bagasse Ash as an Alternative Source of Silicon Dioxide in Sodium Silicate Synthesis

**DOI:** 10.3390/ma16186327

**Published:** 2023-09-21

**Authors:** Jesús A. Pérez-Casas, Antonio A. Zaldívar-Cadena, Anabel Álvarez-Mendez, Juan Jacobo Ruiz-Valdés, Salomé M. de la Parra-Arciniega, David C. López-Pérez, Astrid I. Sánchez-Vázquez

**Affiliations:** 1 Facultad de Ciencias Químicas, Universidad Autónoma de Nuevo León, Avenida Universidad s/n, San Nicolás de los Garza 66455, Mexicojuan.ruizv@uanl.mx (J.J.R.-V.); smdlp@yahoo.com (S.M.d.l.P.-A.); 2 Facultad de Ingeniería Civil, Universidad Autónoma de Nuevo León, Avenida Universidad s/n, San Nicolás de los Garza 66455, Mexico; azaldiva70@hotmail.com (A.A.Z.-C.); david.lopezpr@uanl.edu.mx (D.C.L.-P.)

**Keywords:** sugarcane bagasse ash, sodium silicate, solid state

## Abstract

To reduce the environmental impacts from sodium silicate synthesis, a ceramic method was suggested, with sugarcane bagasse ash (SCBA) as the source of silicon dioxide and sodium carbonate. Although the production of sodium silicate is carried out on a large scale, it should be noted that its process requires temperatures above 1000 °C; it also requires the use of highly corrosive agents such as sodium hydroxide and chlorine gas to neutralize the remaining sodium hydroxide. In the present study, the synthesis temperatures were reduced to 800 °C with a reaction time of 3 h by pressing equimolar mixtures of previously purified SCBA and sodium carbonate; then, heat treatment was carried out under the indicated conditions. The resulting materials were analyzed with Fourier transform infrared spectroscopy (FTIR) and X-ray diffraction (XRD). Among the crystalline phases, calcium disodium silicate was identified, in addition to sodium silicate; thus, it was inferred that the other components of the ash can interfere with the synthesis of silicate. Therefore, in order to obtain the highest composition of sodium silicate, a leaching treatment of the SCBA is required.

## 1. Introduction

As the main residue of the sugar industry, sugarcane bagasse ash (SCBA) has the disadvantage of being accumulated because of the low demand for it. This residue is currently used as fertilizer in sugarcane plantations; however, in Mexico alone, approximately 500 kt of this waste is produced annually. SCBA is not merely an inorganic residue, since it retains carbon in its composition; thus, its accumulation leads to bad environmental practices, such as burning the ash in the open. Considering the high concentration of SiO_2_ in SCBA, its use has been proposed in the synthesis of ceramic materials, mainly light bricks, tiles, and glass-ceramics; however, the problem of the increased porosity in the materials arises, which is explained by the remaining carbon that generates CO_2_ during the synthesis of the materials. When SCBA is used in the synthesis of bricks and tiles, this porosity decreases the mechanical properties, which, in turn, increases the absorption of water, in environments where the temperature reaches ranges that are lower than the freezing point, this can cause the material to collapse [1,2].

In the case of glasses and glass–ceramics, performance problems are caused due to replacing the SCBA with alkaline carbonates that act as fluxing agents. The decomposition of these carbonates and the burning of the remaining carbon in the ash promote the generation of CO_2_ and therefore, pores in the vitreous material. The pores or bubbles in the glass material can decrease if the temperature is increased enough so that the viscosity of the glass allows for the flow of gas, but this implies a high energy consumption [3,4,5].

Instead of synthesizing glass with SCBA, there are other products with diverse applications, such as sodium silicate, which can be used as a binder in concrete, in the synthesis of aerogels for water remediation, as a raw material in cosmetic and paper industries, in the synthesis of high-performance magnets, and in the synthesis of anticorrosive coatings for metals and hydrophobic coatings for ceramics [6,7,8,9,10]. SCBA has the drawback of having a diverse and complex composition; the amount of SiO_2_ is not always the same, and its other components can interfere with the synthesis of glass and glass–ceramics. However, unlike the synthesis of glasses, Na_2_SiO_3_ requires lower temperatures for its synthesis, regardless of whether Na_2_CO_3_ or Na_2_O are used as reagents.

The most common process used in the synthesis of sodium silicate is the thermochemical method, which consists of the reaction of silica with sodium hydroxide (NaOH) at temperatures above 300 °C [11]. However, to isolate SiO_2_ from ashes, the use of HNO_3_ has been reported [12], suggesting an impact on both principles 3 and 6 of green chemistry. Principle number 3 refers to a less dangerous chemical synthesis using chemical substances that are slightly or not toxic to humans and the environment, while principle number 6 regards carrying out designs for energy efficiency, reducing operating costs and environmental impact, and trying to carry out synthesis processes at temperatures close to room temperature [13]. In addition, since the synthesis temperature is lower than the temperature used in other methods, there are some inconveniences with the use of NaOH; the first is that it is a highly corrosive agent that can damage steel alloys [14]; the second is that not all sodium hydroxide reacts during synthesis, so it must be neutralized with chlorine gas; and finally, the use of NaOH represents significant damage to the environment and human health, as reported by Ingrao [15].

In this research, the use of the ceramic method is proposed, which only impacts principle 6 of green chemistry. For the synthesis, pellets were prepared with SCBA, together with sodium carbonate in a 1: 1 molar ratio between silicon oxide and sodium oxide. The use of a carbonate instead of an oxide has some implications, such as stoichiometric calculations, an increase in reaction time because the carbonate must be decomposed prior to the reaction, and the subsequent release of CO_2_ to the atmosphere because of decomposition (Equation (1)). Since the applications of sodium silicate are focused on the solution [9,10,16], the increase in porosity and the mechanical properties of the material obtained are not relevant; thus, in this case, the disadvantages presented of using SCBA have no relevant impact on the result. Since the composition of the ash varies according to geographical location, a characterization is necessary before its use. In addition to this, as it was mentioned before, traces of carbon and other elements were found in the ash; thus, a purification process was necessary.

(1) $${\mathrm{Na}}_{2}{\mathrm{CO}}_{3}\to {\mathrm{Na}}_{2}O+{\mathrm{CO}}_{2}$$

## 2. Materials and Methods

### 2.1. Characterization of Sugarcane Bagasse Ash

The SCBA was obtained from the Xicotencatl sugar mill, located at the south of Tamaulipas, Mexico. In addition, Sigma-Aldrich (99.5%, Wuxi, China) sodium carbonate was used for the synthesis.

The SCBA was analyzed to quantify its content of volatile material, as reported in a previous study [5]. After its analysis, the ash was calcinated, following the procedure reported by Sultana et al. [17]; then, it was subsequently ground by hand in an agate mortar to a homogeneous mesh size of 150 microns to maximize the contact area between the ash and Na_2_CO_3_. The grounded ash was analyzed using XRD (Bruker D2 Phaser, Monterrey, México) to identify the predominant crystalline phase of SiO_2_, and X-ray fluorescence (Panalytical Epsilon 3, Monterrey, México) was used to assess the amount of silicon oxide required to make the corresponding stoichiometric calculations to prepare the pellets with 1:1 and 1:2 SiO_2_–Na_2_O molar ratios. The mixture of reagents was carried out in the same way in an agate mortar to reduce the loss of material in the mills, especially considering the hygroscopic property of Na_2_CO_3_, which makes it easier for it to adhere to the walls of the mill. There is more than one type of sodium silicate structure, and this varies depending on the molar ratio that the reactive oxides maintain, according to the following reaction:

(2) $${\mathrm{XNa}}_{2}O+{\mathrm{SiO}}_{2}\to {\mathrm{Na}}_{2x}{\mathrm{SiO}}_{2+x}$$

As there are other elements in the SCBA, a leaching treatment was necessary. For this process, the ash was leached with 2% wt. citric acid for 2 h at 60 °C, according to the research of Yahya et al. [18], and analyzed using XRF after the leaching process. The impact of this process is reported in the Results and Discussion section.

### 2.2. Sample Preparation

For the preparation of the pellets, a 1 g mixture of SCBA and Na_2_CO_3_ was pressed into a Craver press apparatus with 10 mm dice to obtain the working pressure. Experiments were designed to assess pressures of 2, 6, and 10 tons, and times of 5, 7.5, and 10 min, measuring as a response the integrity of the obtained tablets. Since the measured response is not numerical, the best conditions were those that required less pressure and less time, and allowed the tablet to remain intact.

Once the pellets were obtained, the synthesis was carried out in a Carbolite 1300 W oven (Monterrey, México), at a temperature of 800 °C for 3 h, maintaining the pellets inside the furnace chamber up to room temperature, in order to allow the crystallization to continue in a controlled way.

### 2.3. Sodium Silicate Characterization

The material obtained from pellets with a 1:1 molar ratio was grounded into a fine powder; it was characterized using X-ray diffraction in a Bruker D2 Phaser (Monterrey, México) using Match 3! (version 3.12 Build 214) to identify the crystalline phases, and a Rietveld method to quantify the ratio of the crystalline phase. Samples with a 1:2 molar ratio were analyzed with FTIR in a Bruker Alpha II spectrometer (Monterrey, México) to determine the presence of reagents in the obtained material.

## 3. Results and Discussion

As was reported in our previous study [19], according to the elemental analysis before and after the calcination, a decrease in the %wt./wt. from 15.86 to 0.12% of the carbon content was observed, meaning an almost total elimination. From the XRF analysis (Table 1), it was possible to identify the main inorganic components in the SCBA, in which silicon oxide was the main component, and calcium stood out as the second major component. The high concentration of calcium became relevant, considering that the ionic radii of Ca^2+^ and Na^+^ are very similar (1.14 and 1.16 Å, respectively), which may imply competition in the selectivity of crystallization reactions of silicates [20]. The citric acid leaching process was suggested to reduce the calcium concentration in the ash.

In the pressure test of the SCBA/sodium carbonate pellets, with higher time and pressure, the pellets became fragile. This behavior can be explained due to the increase in pressure; as the pressure between the powder and the dice becomes greater, the friction between the pellet and the walls of the dice becomes greater, culminating in the destruction of the pellet during the procedure of releasing it from the dice [21]. Thus, the best result corresponds to a pressure of two tons, which was the lowest pressure tested, and a time of 5 min. At these conditions, the stress on the pellet was lowest, and the sample was easily removed from the dice.

**Figure 1:**
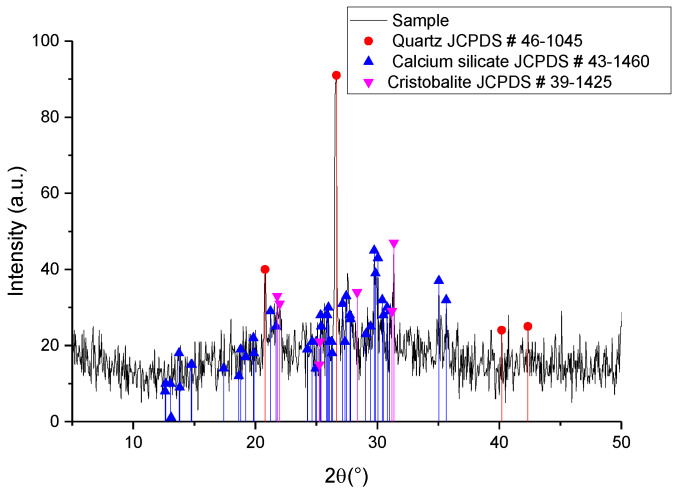
XRD of SCBA after calcination at 900 °C.

Figure 1 shows the diffractogram corresponding to the SCBA, where quartz is observed as the main crystalline phase. This result is different from that reported by Sultana [17], where at a higher temperature the predominant crystalline phase in the SCBA was reported to be cristobalite, after a heat treatment at 800 °C for 2 h. Even so, it could be inferred that the cristobalite formed during calcination reacted with CaO present in the SCBA, forming the calcium silicate observed in the diffractogram. Quartz has an interstitial space in its cell of approximately 3 Å, like the interstitial space of cristobalite [22]. An interstitial space of 3 Å is considered a sufficient distance for the sodium and calcium ions to diffuse through the silicon dioxide crystalline cell. Since calcium silicate appears as a secondary phase, this suggests that other elements react with the silicon dioxide in the ash besides sodium oxide.

**Figure 2:**
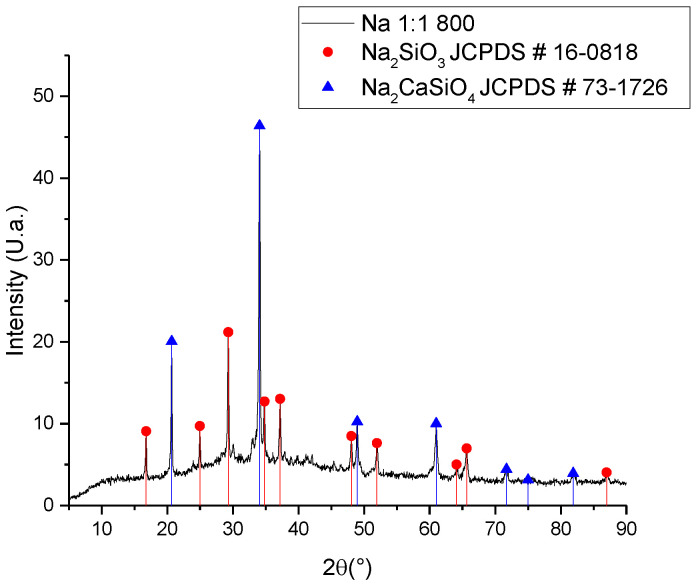
XRD for the sodium silicate synthesized with non-leached ash.

Considering that the reaction was carried out in a solid state, the way it occurs is through a diffusion mechanism between the crystalline cells of silicon dioxide and the other oxide reactants. If the diameter is similar for both ions (sodium and calcium), a kind of competition can be generated to occupy the interstices in the silicon oxide network, thus allowing for the generation of both sodium silicate and sodium–calcium silicate, as shown in Figure 2 [23].

**Figure 3:**
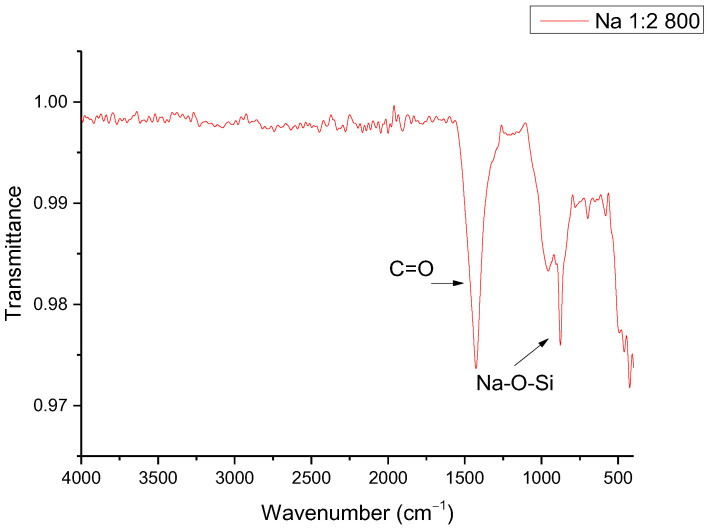
FTIR of sodium silicate synthesized with a molar ratio of 1:2 SiO_2_–Na_2_O.

It is worth mentioning that even though the presence of disodium calcium silicate is an inconvenience for this project, this material is identified as a bioceramic, which has important applications in bone regeneration and localized drug delivery [24]. To increase the ratio of sodium silicate in the synthesized material, samples were synthesized in a molar ratio of 1:2 SiO_2_–Na_2_O; however, little reaction was observed, as shown in the FTIR spectrum of Figure 3. The signal at 1420 cm^−1^ is characteristic of the stretch of the C=O bond present in solid sodium carbonate used as a reagent, suggesting that the lack of reaction can be explained from the thermal stability of the internal carbonate, which requires a higher temperature or a longer synthesis time, considering the heat transfer phenomena to the inner solid material [25,26]. Even so, the presence of the signal at 950 cm^−1^ corresponds to the Na-O-Si bond, suggesting the presence of a chemical reaction [27]. The signals between the range of 500 to 880 cm^−1^ correspond to the bending of the C=O bond outside (600 to 700 cm^−1^) and inside the plane (840 to 880 cm^−1^), while the signal around 1050 to 1100 cm^−1^ is characteristic of the stretching of the Si-O bond [27,28]. The most important signals are those corresponding to 1500 and 950 cm^−1^, because although the signal at 1050–1100 cm^−1^ also corresponds to the stretching of the Si-O bond, it can refer to both the presence of SiO_2_ and some silicate. The signals at the lower wavenumber may correspond to other bonds, such as the bending of the Si-O bond [29].

**Figure 4:**
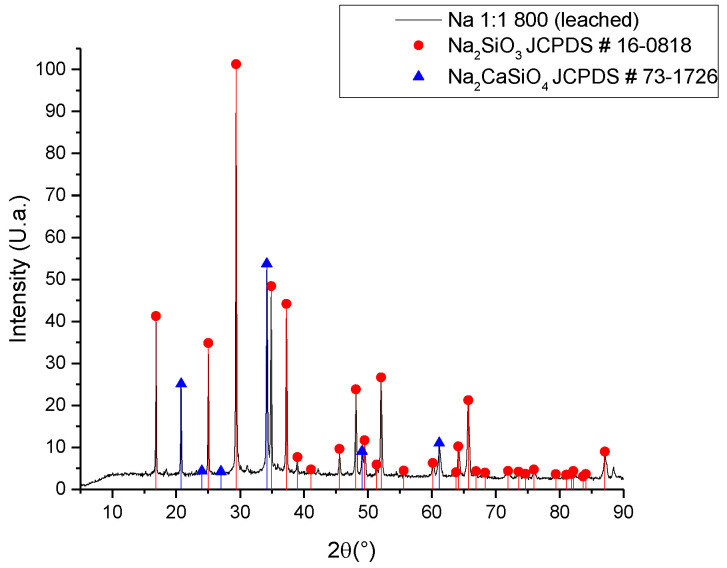
XRD of sodium silicate synthesized with leached ash.

As per the results of the synthesis developed with the untreated ash (Figure 2), the use of a purification technique for the ash was proposed, carrying out a leaching process with citric acid at a concentration of 2% wt./v at a temperature of 60 °C for 2 h [19]. Citric acid, unlike the nitric acid used to extract SiO_2_ from the ashes, is a weak acid with a low environmental impact. In this way, the calcium concentration was reduced, achieving a reduction of approximately 5% of calcium in the ash (Table 1), and obtaining a purer sodium silicate, as observed in the diffractogram of Figure 4.

With sodium silicate as the main crystalline phase, it is possible to separate both silicates, considering that disodium calcium silicate is a water-insoluble compound; thus, a decantation and filtering process can obtain a solution of sodium silicate. Unlike Vinai’s research [11], which used a thermochemical method with NaOH, the results obtained in this research present a single crystalline phase of sodium silicate Na_2_SiO_3_. In Vinai’s report, there are signs corresponding to three polymorphs of sodium silicate. The importance of the homogeneity of the crystalline phases lies in the fact that, in the case of sodium silicate, each crystalline phase has different applications and properties.

Before presenting the results of the Rietveld refinement, it should be considered, in addition to the results presented, that the XRD technique has a detection limit of 5%; thus, the presence of other crystalline phases after heat treatment is possible, such as potassium silicate. In observing what is presented in Table 1, as well as considering the melting temperature of potassium oxide (740 °C) [30,31], it is possible that a synthesis process occurred in parallel.

**Figure 5:**
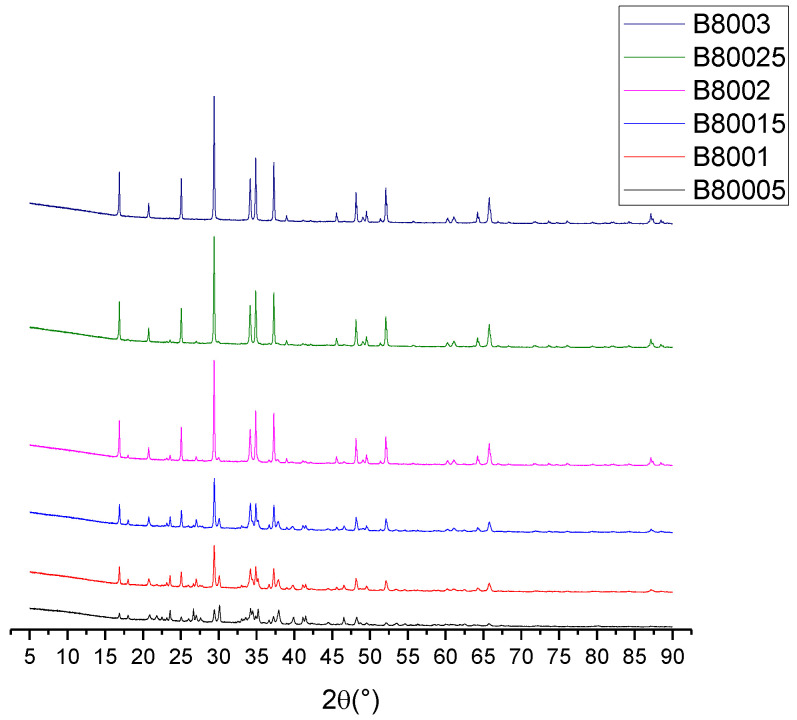
Crystallinity of the sample at different reaction times.

The Rietveld refinement was carried out starting from the results obtained from the XRD analysis, using the software “Match3!” (version 3.12 Build 214), and making use of the Maud software (Version 2.996). For this process, in addition to analyzing the results of the synthesis after 3 h of reaction, the behavior of the reaction was examined from 0.5 to 3 h. Figure 5 shows the diffractograms corresponding to this analysis.

From Figure 5, the sample code is defined by the letter B, which represents the serial experiments, while 800 is the synthesis temperature, which was the same for all of the samples. The last numbers represent the reaction time from 0.5 to 3 h; for example, sample B80005 corresponds to the material synthesized at 800 °C and 0.5 h. It is possible to observe how starting from a diffractogram with a considerable noise content corresponding to amorphous signals coming from the ash, the intensity of the signals becomes increasingly intense until there are no significant differences between 2.5 and 3 h of elapsed time. The Rietveld refinement identified the crystalline phases presented in Table 2. Unlike the other diffractograms presented, the CIF identifications were used instead of the JCPDS, because the Maud software only admitted the crystallographic sheets that corresponded to the CIF files.

Unlike the other diffractograms presented, the CIF identifications were used instead of JCPDS because the Maud software (Version 2.996) only admitted the crystallographic sheets that corresponded to the CIF files. However, the identifications correspond to the same phases identified with their respective JCPDS numbers. Table 3 shows the compositions corresponding to the crystalline phases identified at each reaction time. For the construction of Table 3, the different SiO_2_ polymorphs were integrated into one because when analyzing them individually, confusing information was generated due to the constant change between polymorphs due to temperature; this same behavior was studied by Sultana [17].

The presence of sodium superoxide in the first half hour of the reaction, despite being an unstable crystalline phase under reaction conditions, has been reported as a common intermediate in the formation of Na_2_O from Na_2_CO_3_, according to what was reported by Maheshwari [32]; however, this intermediate disappeared immediately to produce Na_2_O. After 2.5 h of reaction, the presence of SiO_2_ was still observed, which disappeared after half an hour; since the amount of unreacted calcium present is unknown, it is hypothesized that part of the Na_2_CaSiO_4_ decomposes to give rise to Na_2_SiO_3_, while the rest of the SiO_2_ continues to react with the calcium present in the ash. This hypothesis is unfounded, considering that if Na_2_CaSiO_4_ decomposed to produce Na_2_SiO_3_, the amount of Na_2_SiO_4_ would be less than the 19% reported at the end of the experiment.

## 4. Conclusions

Sugarcane bagasse ash can be used in the synthesis of sodium silicate at a lower temperature (800 °C) than that used in the usual synthesis of this compound (1000–1200 °C). However, it is necessary to carry out a purification treatment using citric acid leaching, in order to reduce the calcium concentration, and thus decrease the sodium–calcium silicate concentration.

Increasing the sodium carbonate concentration did not benefit the production of sodium silicate, due to the high thermal stability of sodium carbonate inside the pellet, which was the reason for using a 1:1 molar ratio between SiO_2_ and Na_2_O. Carrying out the synthesis with SCBA treated with citric acid resulted in Na_2_SiO_3_ as the main product, reaching up to 81% of the composition of the final product. To purify the product obtained, it is possible to make use of the insolubility of Na_2_CaSiO_4_.

## Figures and Tables

**Table 1:** SCBA composition after calcination and purification.

Compound	%wt./wt.	Leached Ash %wt./wt.
Na_2_O	0.48	0.00
MgO	0.92	0.30
Al_2_O_3_	2.21	1.70
SiO_2_	70.85	78.61
P_2_O_5_	1.15	1.28
SO_3_	1.46	0.25
K_2_O	4.34	5.61
CaO	12.73	7.45
TiO_2_	0.44	0.47
MnO	0.13	0.11
Fe_2_O_3_	4.82	4.05
CuO	0.03	0.02
ZnO	0.06	0.03
SrO	0.13	0.05
ZrO_2_	0.02	0.01
Ag_2_O	0.18	0.06
BaO	0.04	0.00
Total	100	100

**Table 2:** Crystalline phases identified for Rietveld refinement.

Phase	CIF
Na_2_CO_3_	96-210-6298
Na_2_SiO_3_	96-231-0859
Na_2_CaSiO_4_	96-101-0112
Quartz	96-901-5023
SiO_2_ (no polymorph identified)	96-900-6299
NaO_2_	96-412-4632
Cristobalite	96-901-4487

**Table 3:** Composition of the sample taken at different reaction times.

Sample	SiO_2_	Na_2_CO_3_	NaO_2_	Na_2_SiO_3_	Na_2_CaSiO_4_
B0.5	0.42	0.31	0.10	0.12	0.045
B1.0	0.39	0.27	0.00	0.27	0.072
B1.5	0.34	0.27	0.00	0.28	0.10
B2.0	0.23	0.20	0.00	0.44	0.13
B2.5	0.24	0.00	0.00	0.62	0.14
B3.0	0.00	0.00	0.00	0.81	0.19

## Data Availability

Not applicable.
